# Assessment of the Mechanical Performance of AlCrSiN Coating Implanted with Zr and Ta Ions

**DOI:** 10.3390/ma19030569

**Published:** 2026-02-02

**Authors:** Jing Liang, Laia Ortiz-Membrado, Raul Bonet, Jordi Orrit-Prat, Jaume Caro, Jonathan Fernández de Ara, Eluxka Almandoz, Qingdong Ruan, Ricky King-Yu Fu, Paul K. Chu, Luis Llanes, Emilio Jiménez-Piqué

**Affiliations:** 1 CIEFMA—Department of Materials Science and Engineering, EEBE, Universitat Politècnica de Catalunya-BarcelonaTECH, Avda. Eduard Maristany 16, 08019 Barcelona, Spainluis.miguel.llanes@upc.edu (L.L.); 2 Barcelona Research Center in Multiscale Science and Engineering, Universitat Politècnica de Catalunya-BarcelonaTECH, Avda. Eduard Maristany 16, 08019 Barcelona, Spain; 3 Eurecat, Centre Tecnològic de Catalunya, Unit of Metallic and Ceramic Materials, Plaça de la Ciència 2, 08243 Manresa, Spain; raul.bonet@eurecat.org (R.B.); jordi.orrit@eurecat.org (J.O.-P.); jaume.caro@eurecat.org (J.C.); 4 Centre of Advanced Surface Engineering, AIN, 31191 Cordovilla, Spain; jfernandez@ain.es (J.F.d.A.); ealmandoz@ain.es (E.A.); 5 Science Department, Universidad Pública de Navarra (UPNA), Campus de Arrosadía, 31006 Pamplona, Spain; 6 Department of Physics, City University of Hong Kong, Tat Chee Avenue, Kowloon, Hong Kong 999077, China; 7 Department of Materials Science and Engineering, City University of Hong Kong, Tat Chee Avenue, Kowloon, Hong Kong 999077, China; 8 Department of Biomedical Engineering, City University of Hong Kong, Tat Chee Avenue, Kowloon, Hong Kong 999077, China

**Keywords:** AlCrSiN coating, ion implantation, mechanical performance

## Abstract

This study explores the impact of Zr and Ta ion implantation on the mechanical performance of an AlCrSiN quaternary coating deposited on a WC-Co cermet substrate. Nanoindentation tests revealed a decrease in hardness and elastic modulus after ion implantation, compared to unimplanted coatings. Moreover, microscratch and contact damage tests demonstrated improved adhesion and reduced surface damage for ion-implanted samples, with Ta implantation exhibiting the best performance. Scanning Electron Microscopy (SEM) and focused ion beam (FIB) cross-sectional analysis confirmed less severe damage in ion-implanted samples compared to unimplanted ones. These findings suggest that Zr/Ta ion implantation enhances the structural integrity and adhesion of AlCrSiN coatings under loading conditions commonly encountered in practical applications, despite a moderate reduction in intrinsic hardness and elastic modulus.

## 1. Introduction

Hardmetals are materials composed of a hard WC phase bonded by a metal alloy, generally cobalt, and are widely used in industrial tools like forming, cutting, and milling due to their exceptional mechanical properties, exhibiting high hardness and high fracture toughness, which result in a high wear resistance [1,2,3] and mechanical reliability [4,5,6]. High-performance cutting tools make full use of the advantageous properties of hard metals and increase the service life of cutting tools by depositing hard ceramic coatings [7,8].

Quaternary coating systems, like AlCrSiN, AlTiSiN, and AlCrBN, are based on traditional metal nitride ceramic coatings (such as CrN and TiN) with the introduction of other elements to improve the mechanical properties, especially hardness, thermal stability, oxidation resistance, and wear resistance [9,10,11]. AlCrSiN coating is one of the most used coatings to protect cutting tools and further to expand the service life under severe working conditions [12]. The structure of the AlCrSiN coating obtained by magnetron sputtering consists of (Al,Cr)N columns with a small amount of amorphous SiN_x_ at the column boundaries, which inhibits the growth of crystalline (Al,Cr)N and enhances the hardness of the coating [13,14].

Ion implantation has been proven to be an effective technique for improving the mechanical properties of cemented carbide materials and PVD hard coatings deposited on them. For example, in a study by Shum et al. [15], TiAlN coatings were deposited on pre-treated Ti-implanted WC-Co substrates. The formation of the TiC alloy phase resulted in an increase in the hardness of the implanted substrate, which improved the adhesion and mechanical support of the TiAlN coating, as well as the performance in a cut test. Similarly, in another study by Fu et al. [16], N, Mo, and Mo/W implantation was applied to ternary carbide WC-TiC-Co materials, resulting in improved hardness and wear resistance. This was achieved through the formation of tungsten nitride, titanium nitride, and molybdenum carbide in the WC-TiC grains in a solid-state precipitate phase or nanoclusters. In addition, Ortiz et al. [17] also studied the effect of Ti and Cr ion implantation in WC-Co substrates coated with AlCrSiN. As a result, the adhesion strength of AlCrSiN coatings was improved due to a synergic enhancement of the fracture toughness and load-bearing capability of the substrate.

Ion implantation has been extensively applied to TiN PVD hard coatings. Early work of Mitsuo et al. demonstrated the improvement of high-temperature oxidation resistance of TiN coatings by Al implantation due to the formation of Al oxides [18]. Wang et al. [19] have demonstrated that AlN and/or ternary TiAlN are formed as a result of Al ion implantation or TiN. Carbon implantation of TiN has been found to increase the hardness in the near-surface region, owing to the formation of TiC and TiCN. Additionally, the friction coefficient is considerably reduced by the formation of a carbonaceous layer on the surface, which serves as a solid lubricant [20]. In a study by Deng et al. [21], similar results were observed with the Nb and C implantation of TiN, where the formation of NbN, Nb_2_O_5_, and TiC phases resulted in an increase in hardness due to the strain hardening effect caused by lattice mismatch. Similarly, vanadium implantation of TiN coatings led to the generation of hard VN and TiVN compounds [22]. Zr implantation, on the other hand, has been shown to enhance the corrosion resistance of TiN in saline environments [23]. This protection is attributed to the closure of existing pinholes and the formation of various nitrides, oxides, and oxynitrides of Ti and Zr. Finally, the work by Tian et al. [24] has shown that high-dose W implantation of TiN induces a remarkable decrease in the friction coefficient, due to the formation of lubricious tungsten and titanium oxides.

CrN coatings have also been the subject of considerable interest. Weng investigated the effects of C and V implantation on CrN coatings and found that compared to un-implanted samples, ion implantation led to densification of the film, increased compressive residual stress, and hardness, resulting in improved wear and corrosion resistance [25,26]. Furthermore, Nb, Zr, and Cr implantation has been shown to enhance the corrosion resistance of CrN coatings by promoting the formation of different binary and ternary nitrides, oxides, and oxynitrides at the surface [27,28,29].

The investigation of ion implantation on ternary nitride hard coatings is limited. Boron implantation into TiAlN, for instance, enhances the tribological properties of the coating by producing TiB_2_ and BN phases [30]. The improved wear resistance is attributed to the hardening effect of TiB_2_, while the lubricious effect of BN leads to a decrease in the friction coefficient. Similarly, Nb implantation on TiAlN coatings improves the wear resistance due to the formation of an amorphous and nanocrystalline structure [31]. Moreover, Nb implantation in TiAlN/CrN nanomultilayer coatings produces NbN and Nb_2_O_5_, and a small amount of niobium oxynitride in the implanted zone [32]. This results in an increase in hardness and a decrease in friction coefficient due to the amorphous top layer on the surface that acts as a solid lubricant during wear sliding.

In contrast, there is a dearth of research on ion implantation of quaternary nitride hard PVD coatings. Chang et al. reported the advantages of Mo and C implantation of CrAlSiN coatings in high-temperature wettability by molten glass [33]. Mo carbides were found in the implanted layer of CrAlSiN, while the compressive stress increased by more than two times. The wettability of the implanted films by molten glass at 500 °C decreases due to the presence of an oxide layer consisting of molybdenum, chromium, and aluminum oxides on the surface. This makes the films resistant to inter-diffusion between the glass and the coating.

Despite the fact that it has been demonstrated that AlCrSiN coatings deposited on WC-Co substrates improve mechanical performance, the impact of ion implantation has not yet been studied [34]. Therefore, the objective of this study is to understand the effect of ion implantation of Zr and Ta ions on the mechanical properties of quaternary AlCrSiN coatings.

## 2. Materials and Methods

### 2.1. Sample Preparation

A WC-Co hardmetal, with 0.9 ± 0.4 µm of grain size and 10% of cobalt content, coated with an AlCrSiN PVD layer with a disk of Φ 30 mm × 3 mm, was selected for this study. Cathodic arc evaporation was used to deposit the AlCrSiN coating. The process was carried out using the industrial equipment Platit π80 units (Platit, Selzach, Switzerland) in a vacuum chamber with an argon atmosphere at 0.8 × 10^−2^ Pa and a negative bias voltage of −65 V. Incorporating the LARC technology (lateral rotating cathode) and using pure Cr at 99.9% and Al + 12% at Si as material sources, a ~0.8 μm single-layered nanocomposite AlCrSiN coating was deposited [17].

Zr and Ta were implanted in AlCrSiN coatings by means of the Metal Vapor Vacuum Arc (MEVVA) ion implantation technique. Zr and Ta cathodes (99.95% purity) were evaporated using a pulsed metal cathodic vacuum arc source (MEVVA50, Plasma Technology Limited, Hong Kong, China). In all processes, the vacuum chamber was pumped down to a pressure below 5·10^−4^ Pa before conducting the ion implantation. The samples were placed on a water-cooled and grounded substrate holder. The acceleration ion implantation voltage was 50 kV, and the mean beam current was about 5 mA. The treatment time was 4 h. Taking into account that the sample holder area was 200 cm^2^ and the ion beam characteristic in the MEEVA source, the incident ion dose was estimated to be in the range of ~2.3–2.6 × 10^17^ ions/cm^2^.

### 2.2. Chemical Composition Analysis

X-Ray Photoelectron Spectroscopy (XPS) measurements were performed in a PHI VersaProbe II from Physical Electronics (Chanhassen, MN, USA). The source of X-rays was Al (1486.6 eV) monochromatic at 25.1 W, with a beam diameter of 100 μm. The samples were sputtered with 1.0 kV Ar^+^ monoatomic, 1 presputter cycle, 60 sputter cycles, alternating Zalar sputter mode, with sample rotation and a sputter rate of 3.1 nm/min in SiO_2_.

### 2.3. Nanoindentation and Microindentation

An MTS nanoindenter XP (MTS System, Eden Prairie, MN, USA) equipped with a continuous stiffness measurement (CSM) module was used to perform indentations at a constant rate strain (0.05 s^−1^) with a Berkovich tip calibrated using fused silica. A total of 25 indentations were conducted on the surface of all samples with a maximum penetration depth of 2000 nm. The separation distance was kept at 50 µm to avoid the overlap between neighboring indentations. The results were analyzed by the Oliver and Pharr method [35]. As the CSM technique provides a continuous measurement of hardness and Young’s modulus, it allows for measuring these properties at any penetration depth. The continuous measurement allowed us to measure hardness and Young’s modulus independent of the substrate. The intrinsic hardness value of the coating was obtained by measuring the hardness at a penetration depth around 1/10 of the thickness of the coating with a maximum normal load of 490 mN [36,37,38]. The elastic modulus was calculated by extrapolating the results to zero depth, as recommended by ISO 14577 [39].

Vickers hardness was measured by the Vickers Testwell FV-700 (FUTURE-TECH CORP.,Kanagawa, Japan). The average and standard deviation for each sample under five different loads (9.8 N, 49 N, 98 N, 196 N, and 294 N) were calculated by the following:

*H_V_* = 1.854P⁄d^2^ (1)

where P is the applied load and d is the diagonal of the projection of the imprints.

### 2.4. Adhesion

Adhesion strength was evaluated by scratch and contact damage tests. For a scratch test, a Rockwell C diamond tip was used, with a scratch length of 5 mm and an increasing load from 0.9 N to 120 N, at a constant rate of 10 N/min. The first appearance of failure was cohesive failure, labeled (*L_c_*_1_), and the second was adhesive failure, labeled (*L_c_*_2_), following the standard ASTM-C1624 [40]. Damage was observed by SEM.

For the contact damage test, a Rockwell C tip was applied to the surface with a constant load in order to see deformation, cracks, and spallation [41]. Five loads—98 N, 196 N, 392 N, 613 N, and 980 N—were used to create different damage scenarios.

### 2.5. Microscopy

Images were obtained by optical microscopy and SEM, using a PhenonXL (Phenom-World BV, Eindhoven, The Netherlands) and a SEM/FIB (Carl Zeiss AG, Baden-Württemberg, Germany), respectively. Cross sections were obtained by Focus Ion Beam (FIB) using decreasing currents for milling down to 500 pA as the final polishing procedure. A platinum layer was deposited on the position of interest to prevent the waterfall effect during milling processing. The confocal laser scanning microscope (CLSM) (Olympus FV3000, Yokohama, Japan) uses a laser as a scanning light source to scan the image point by point.

## 3. Results and Discussions

### 3.1. Analysis of the Implanted Ions by XPS

**Figure 1:**
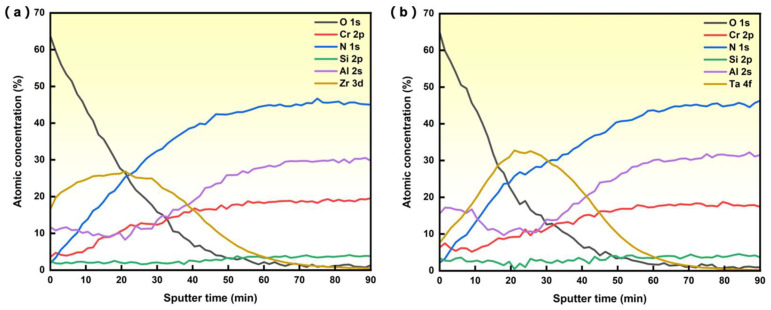
XPS depth profile of atomic concentration of AlCrSiN coating with Zr implantation (**a**) and Ta implantation (**b**).

Figure 1a,b show the atomic concentration as a function of Ar sputter time of AlCrSiN coatings ion-implanted with Zr and Ta, respectively. As was to be expected, the concentration profile of the implanted ions shows a Gaussian-like distribution, with a maximum concentration of 26.5% of Zr and 32.5% of Ta. Table 1 shows the surface element concentration of AlCrSiN coatings with and without ion implantation.

### 3.2. Characterization and Mechanical Properties of the Coated Materials

The thickness of the coating was measured by the SEM images of the cross-section after FIB. The roughness of the surface was measured by the confocal microscope, as presented in Table 2, where RSa is the arithmetical mean height of the surface and RSz is the maximum peak-to-valley height.

The hardness of AlCrSiN coatings with ion implantation presents lower values (~22 GPa) than the samples without ion implantation (~34 GPa), as presented in Table 3. The trend of hardness through ion implantation is consistent with the conclusion of previous studies that hardness is generally lowered by ion implantation, attributed to the distortion formed in the crystalline phase at the surface of the material after ion implantation [42,43]. The elastic modulus of the sample without ion implantation is 553 GPa, while in the implanted samples the elastic modulus decreased significantly.

**Figure 2:**
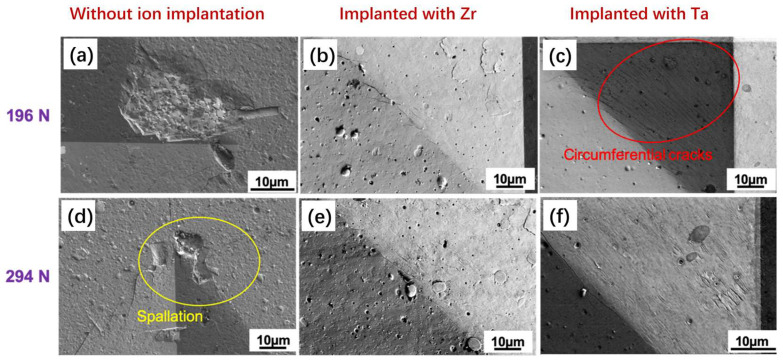
Details of the Vickers imprints of the different materials at 196 N and 294 N. Spallation is clearly seen on the unimplanted coating, while the ion-implanted coatings only present narrow microcracking. (**a**,**d**) The sample without ion implantation under 196 N and 294 N, respectively. (**b**,**e**) The sample implanted with Zr under 196 N and 294 N, respectively. (**c**,**f**) The sample was implanted with Ta under 196 N and 294 N, respectively.

The Vickers hardness measured at 1 kg of AlCrSiN coatings without ion implantation was about 21 ± 1 GPa. For the sample with Ta ion implantation, the value was 18 ± 0.5 GPa, and for the case of the Zr ion implantation, it was 16 ± 0.4 GPa measured at 1 kg. It has to be taken into account that the deformation under the Vickers loads is also governed by the influence of the substrates. In Figure 2, details of the indentation imprints produced by Vickers indentation are presented. It can be seen how the sample without ion implantation produces spalling of the coating, while the indentations on the samples with ion implantation only show a small amount of microcracking.

### 3.3. Adhesion Test

**Figure 3:**
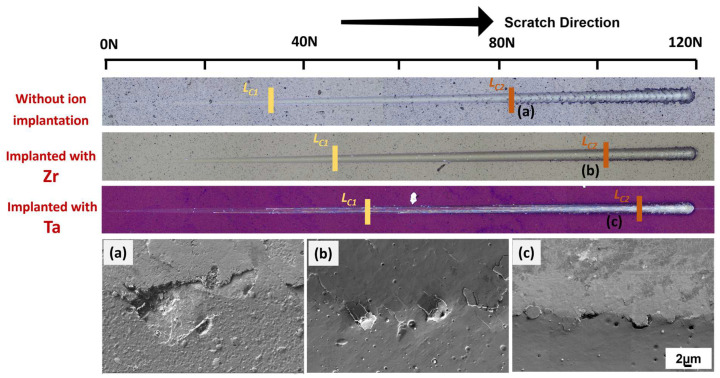
Adhesion optical profile and SEM image of failure of AlCrSiN coatings after microscratch tests. The position of *L_C_*_2_: (**a**) without ion implantation, (**b**) implanted with Zr, and (**c**) implanted with Ta.

Figure 3 shows the optical images of the scratch tracks and magnified failure events as observed by SEM for the three materials. The critical loads, *L_c_*_1_ and *L_c_*_2_, are also indicated in the figure. *L_c_*_1_ is the first critical load and corresponds to cohesive failure characterized by the formation of ring cracks on the coatings. At higher loads, the second critical load, *L_c_*_2_, is reached. This critical load corresponds to adhesive failure of the coating, resulting in spalling and delamination. The failure mode for samples implanted with Zr and Ta showed similar failure loads and fracture surfaces, as shown in Figure 3b,c, respectively. In both cases, the loads were significantly higher than the sample without ion implantation, indicating that ion-implanted samples exhibit better resistance under a progressive sliding normal load. Additionally, the spalling of the sample without ion implantation was more severe than the samples with ion implantation, as shown in Figure 3a, suggesting better fracture resistance. Among the ion-implanted samples, the Ta-implanted sample showed the highest resistance to scratch.

**Figure 4:**
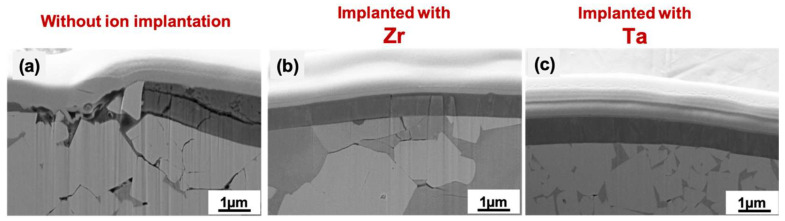
Cross-section SEM images of a scratch about 90 N of AlCrSiN coating, (**a**) without implantation, (**b**) implanted with Zr, and (**c**) implanted with Ta deposited on WC-Co substrate.

FIB cross sections were conducted to observe the damage resulting from scratch testing at the location where a load of 90 N was applied, inducing damage for all samples. Figure 4 shows the SEM images of the cross sections. The reference coating exhibits extensive cracking in both the coating and substrate, along with evident delamination. In contrast, the Zr-implanted coating displays some cracking in the substrate, but the coating surface did not exhibit cracking, and incipient delamination was observed. Furthermore, in the case of the Ta-implanted coating, very minimal damage was observed in the substrate, and the coating maintained its structural integrity. As a result, the AlCrSiN coating implanted with Ta demonstrated better adhesion.

**Figure 5:**
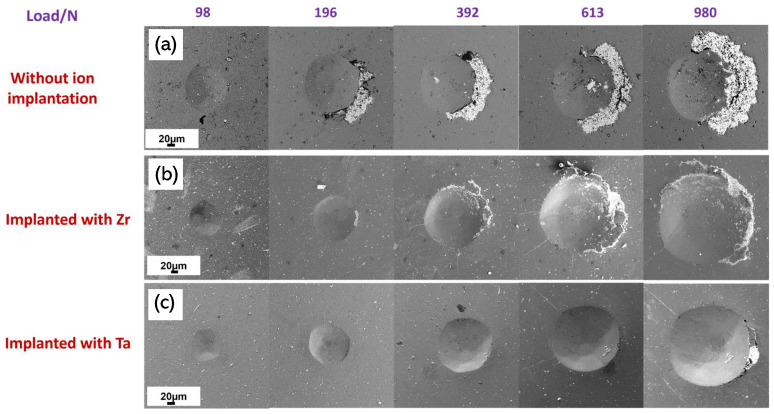
SEM of Contact damage test at different loads with Rockwell C tip of AlCrSiN coatings deposited on WC-Co substrates without ion implantation (**a**) and implanted with Zr (**b**) or Ta (**c**).

A contact damage test was performed to confirm the improved adhesion of the ion-implanted coatings. Figure 5 shows the results of the test, where the untreated coating exhibited delamination starting at a load of 196 N. On the other hand, the coatings with Ta ion implantation showed no apparent damage at that load. However, for the sample with Zr ion implantation, a small area of coating delamination was observed to the right of the footprint, which was much smaller compared to the sample without ion implantation under the same loading conditions. The delamination for both ion treatments started at higher loads, and the area was much smaller. In the case of Ta ion-implanted coating, delamination was only observed at 980 N and with a very small surface area. These results are consistent with the previous result of the scratch test. Additionally, in all samples, chipping phenomena of the coating were formed on the right side of the footprints, which would indicate a possible misalignment of the indenter with respect to the sample.

**Figure 6:**
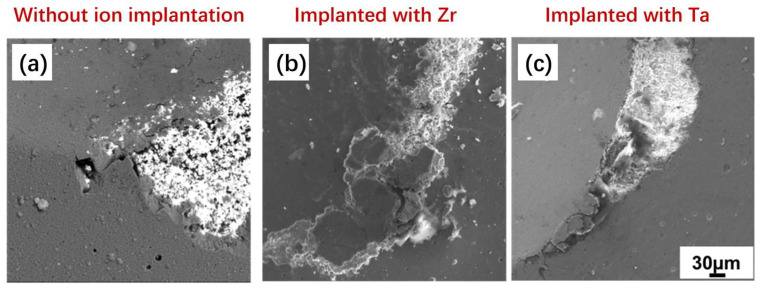
SEM of contact damage test at 980 N with Rockwell C tip of AlCrSiN coatings without ion implantation (**a**) and implanted with Zr (**b**) or Ta (**c**).

To further observe the failure after 980 N, images obtained by SEM are presented in Figure 6. The WC-Co substrate was exposed for all samples, but the coating remained more adhered for the samples with ion implantation, confirming the increased adhesion strength of the coating after ion implantation.

Using the results obtained from scratch testing, the critical stress for spallation, $\sigma_{c}$, was calculated by [44,45] the following:

(2) $$\sigma_{c}=\left(\frac{2L_{c2}}{\pi d_{c}^{2}}\right)[\frac{\left(4+\nu_{f}\right)3\pi \mu }{8}-1+2\nu_{f}]$$

where *d_c_* is the width of the track at *L_c_*_2_, μ is the friction coefficient obtained from the scratch test, and *ν_f_* is the Poisson rate of coatings, equal to 0.25, followed by [8,46]. The surface energy of the interfacial crack is known and defined by Equation (3) as follows:

(3) Gc=σc2t2Ef

where *t* is the thickness of the coating, and the *E_f_* is the elastic modulus of coatings.

Table 4 displays the value of critical load *L_c_*_1_ and *L_c_*_2_ obtained from the optical images, as well as the critical stress ($\sigma_{c}$) of coatings delaminated or spalled, from the substrate, and interfacial fracture energy (*G_c_*). The sample of AlCrSiN implanted with Ta exhibits the highest adhesive energy with a value of *G_c_* = 247 J/m^2^ and a critical stress of $\sigma_{c}$ = 5.01 GPa, which is in agreement with the results obtained from the crack propagation resistance (CPRs) microscratch test.

The values of *CPRs* after scratch were calculated by *L_c_*_1_ and *L_c_*_2_ is [47]:

(4) $$CPRs=L_{c1}\left(L_{c2}-L_{c1}\right)\;$$

where *L_c_*_1_ is the lower critical load, and *L_c_*_2_ is the higher critical load.

Specifically, the sample implanted with Ta showed significantly better crack resistance than the one implanted with Zr. This enhanced performance can be attributed to the softening of the material, which allows for better accommodation of deformation under contact loading and scratch testing. In contrast, untreated material must accommodate deformation through cracking and spalling of the coating.

## 4. Conclusions

In this study, the effect of Zr and Ta ion implantation on AlCrSiN coating deposited on a WC-Co substrate is investigated. The hardness and elastic modulus of the coating decreased by 38.3% and 11.0% for the sample implanted with Zr, by 33.8% and 16.6% for the sample implanted with Ta, compared with the sample without ion implantation. The values are consistent with previous studies that have reported a degradation of mechanical properties after ion implantation. Microscratch and contact damage tests demonstrated that AlCrSiN coating with Zr or Ta ion implantation exhibited better adhesion, with Ta ion implantation showing the best performance because of the best critical stress and interfacial fracture energy. SEM and FIB observations confirmed that the samples without ion implantation suffered more severe damage. In summary, the ion implantation treatment of AlCrSiN quaternary coatings improved their mechanical performance and structural integrity under loading conditions, such as scratch and contact loading, that are commonly encountered in service.

## Figures and Tables

**Table 1:** Surface element concentration of AlCrSiN coatings with and without ion implantation.

Sample	Element Concentration (at.%)							
	Al_2s_	Cr_2p_	Si_2p_	N_1s_	O_1s_	Zr_3d_	Ta_4f_	C_1s_
Without ion implantation	14.61	17.02	2.50	27.29	11.25	---	---	27.33
Implanted with Zr	10.53	3.65	1.83	2.30	54.80	17.82	---	9.07
Implanted with Ta	15.00	5.94	2.61	2.34	53.43	---	10.63	10.06

**Table 2:** Thickness and roughness of coated samples.

No.	Coating: AlCrSiN		Thickness (µm)	Roughness (µm)	
	Substrate	Implanted Ion		RSa	RSz
S1	WC-Co	--	0.88 ± 0.10	0.048 ± 0.003	4.9 ± 0.6
S2	WC-Co	Zr	0.72 ± 0.07	0.035 ± 0.003	2.6 ± 0.7
S3	WC-Co	Ta	0.92 ± 0.08	0.044 ± 0.002	3.5 ± 0.2

**Table 3:** Mechanical properties of coated samples.

No.	Coating: AlCrSiN		Hardness (GPa)	Elastic Modulus (GPa)
	Substrate	Implanted Ion		
S1	WC-Co	--	34.0 ± 5.0	553 ± 55
S2	WC-Co	Zr	21.0 ± 0.5	492 ± 64
S3	WC-Co	Ta	22.5 ± 0.4	461 ± 82

**Table 4:** Critical load and adhesion energy of the coated system.

No.	Coating: AlCrSiN		$L_{c1}$ (N)	$L_{c2}$ (N)	$\sigma_{c}$ (GPa)	Gc (J/m^2^)	CPRs (N^2^)
	Substrate	Implanted Ion					
S1	WC-Co	--	33	81	3.04	69 ± 6	1683
S2	WC-Co	Zr	46	102	3.03	67 ± 7	2576
S3	WC-Co	Ta	52	109	5.01	247 ± 24	2964

## Data Availability

The original contributions presented in this study are included in the article. Further inquiries can be directed to the corresponding author.
